# Storage, Retention, and Use of Leftover Pathology Specimens: The Underestimated Treasures

**DOI:** 10.7759/cureus.53025

**Published:** 2024-01-26

**Authors:** N. Fazulunnisa Begum, Karthikeyan Ramalingam, Pratibha Ramani

**Affiliations:** 1 Oral Pathology and Microbiology, Saveetha Dental College and Hospitals, Saveetha Institute of Medical and Technical Sciences, Saveetha University, Chennai, IND

**Keywords:** retrieval, biopsy, specimen analysis, specimen handling, proper specimen storage, pathology, archives, left out specimen, retention, storage

## Abstract

The proper regulations for storage, retention, and use of archived specimens in pathology laboratories and academic institutions are yet to be established. These specimens could be used appropriately for research purposes. Ideal storage and retention in a controlled environment is necessary, and there is a lack of established rules regarding the ownership of the tissue specimens, paraffin blocks, and slides. Though there are numerous uses of formalin-fixed tissue specimens, blocks, and slides, there are also problems in archiving them that hinder their use. This review article addresses the above issues and proposes simple guidelines for the effective use of archived specimens.

## Introduction and background

The practice of a pathologist involves not only the procurement of biopsy specimens and providing histopathological diagnosis but also plays a vital role in storing specimens, tissue blocks, and slides for different service requirements of clinicians, patients, and researchers [1,2]. Pathology departments of the parent institution are the most common storage centers for surgical specimens. Pathologists and their department archives are an abundant source of information and knowledge that can be widely used for research in revealing the origin and expression of human disease [3].

Archives in the pathology department or laboratories are minimally accessed. It comprises formalin-fixed tissues, formalin-fixed paraffin-embedded (FFPE) blocks, and slides arranged in sequential order with the aim of easy retrieval at the patient's request or for research purposes. However, ambiguity exists in handling human tissue specimens after the histopathological diagnosis, and the use of archival specimens in pathology research is an additional challenge due to the lack of standard guidelines addressing their use and the requirement of informed consent. Ownership over remaining specimens after histopathological diagnosis remains unclear [1,2]. This is relative to local legislation and not a universal truth. In most countries, the patient and his/her relatives have ownership, and the archive is only stored until the patient wants such for consultation and further testing. In general, a period of 10-20 years is a lower-limit guideline for retention. Major institutions prefer to store their slides and tissue blocks for 10 years, whereas cancer referral centers preserve them for 25 years [1].

Organizations use different protocols to retain leftover tissue specimens, blocks, and slides. Insufficient space for storage and management issues are frequently encountered. Though the department of pathology/pathology laboratory is an ideal location to collect the specimens and deliver the reports to clinicians and patients, the neglect in specimen storage, space management, and activity planning has caused many hitches to the laboratories [2].

The use of human tissues and their derivatives dispersed among pathology laboratories, museums, and archives has become a contentious topic for courts. Conflicts emerged over the use of human tissue in research and overlapping claims, which occurred when several parties wanted ownership of the same clinical material. A few nations had created legislation governing the use of human tissues. For instance, the United Kingdom (UK) has The Human Tissue Act of 2004 in effect, while the United States (US) has The Common Rule under the Code of Federal Regulations governing the use of human tissue and safeguarding research subjects. The Indian Council of Medical Research (ICMR) has established ethical principles for biomedical research involving human subjects. However, regulating the use of human tissues requires further clarification [1].

In this review, the authors have attempted to propose new guidelines for the proper storage and retention of leftover specimens, blocks, and slides for appropriate use without any ethical, legal, or ownership issues.

## Review

Storage and retention of pathology specimens

Specimen retention is the length of time various specimens are to be retained and monitored. Routinely, they are not kept for a longer time than necessary. Specimens can be retained for repeat or additional testings when needed, new test validation for investigations for public health, and quality control purposes. Generally, the lower limit guideline for the retention period is 10-20 years [4].

In India, the major institutions preserve slides and blocks for around 10 years, and cancer referral centers preserve them for 25 years. College of American Pathologists (CAP) and the Joint Commission on Accreditation of Health Care Organizations in the US recommended that the paraffin blocks, wet tissues, histology slides, and cytology smears should be retained for 10 years and the peripheral smears for seven days or until the appropriate patient care [5]. The Royal College of Pathologists in the UK recommended the preservation of the blocks permanently, and the retention of the slides and smears for at least 10 years and the wet tissues for around four weeks after the report is delivered. The ethical and legal acceptability of continued storage must be reviewed, where material storage is no longer required for clinical purposes but is desirable for teaching, quality assurance, audit, research, or other purposes of public benefit. It is nevertheless good practice, when practical, to check that the patients have not lodged a specific objection to such use during the normal consent processes for the procedure(s) they have undergone [6].

Leftover samples are the residue or the remains of a sample that has been obtained and used for clinical purposes, and would otherwise be discarded [7]. After diagnosis, the leftover specimens are stored in 10% neutral buffered formalin, the volume of which should be 20 times more than the volume of the specimen itself [8]. According to the National Cancer Institute (NCI) Best Practices for Biospecimen Resources, the paraffin blocks and the slides should be stored in 30-70% humidity, at a temperature less than 27°C, and with control systems for parasite infestation and flood risk. However, given the CAP requirement of retaining FFPE tissue blocks for at least 10 years, many laboratories indicated that they would terminate block storage and destroy blocks upon meeting this 10-year retention requirement [9]. The slides are to be placed away from any direct light to preserve stain intensity. The unstained slides are to be stored in dry conditions to prevent hydrolysis and the preservation of proteins for immunohistochemistry, and molecular hybridization [10].

Uses of formalin-fixed tissues and blocks

Specimen preservation can be best done either by using fixative solutions or by using paraffin-embedded blocks. Archival tissue preservation stabilizes the specimen and preserves it for years without deteriorating. These have a vital role in patient care, in the evaluation of diagnosis of recurrent cases, as an educational tool, for biomedical and diagnostic research purposes, as a specimen reserve bio-bank for further molecular studies, and as a storage of rare disease cases [11]. Under the Final Rule published in the Federal Register on January 17, 2017, studies involving deidentified biospecimens and data are not considered human subjects research; therefore, these studies are not regulated by the Common Rule [9].

FFPE tissue samples are a good source of DNA expression information and are not much used because of the degradation and modification of RNA. Microarray analyses can monitor gene expression from biological samples, which can help reveal unrecognized genes. Marshall et al. conducted a study that retrospectively analyzed the archived pathology specimens for RNA extraction. They reported that though formalin-fixed tissues yielded a compromised RNA and transcriptional information was lost, it was still suitable for the Affymetrix GeneChip Analysis (Applied Biosystems, Massachusetts, US), and major pathways were also identified using Ingenuity Pathway Analysis (QIAGEN Silicon Valley, California, US). They proved the use of archived formalin-fixed tissues for gene expression profiling [12]. Roberts et al. used a random primer-based RNA amplification strategy and found similar results obtained from frozen and FFPE samples. Thus, the archived specimens could be used for numerous research procedures [13,14].

Morphologic studies of stored specimens and histopathological studies of archived hematoxylin and eosin-stained slides could be carried out. Immunophenotypic assessment with histochemistry and even cytogenetic procedures on DNA and RNA could be performed with FFPE samples. Formalin fixation causes the degradation of nucleic acids; hence, the detection of clonally rearranged antigen receptors or related genes in polymerase chain reaction (PCR)-based clonality assays is difficult [14]. Varied fields utilizing archived specimens in laboratories are transcriptome and gene expression analysis, multiplex immunofluorescence for spatial signatures, epigenetic profiling, spatial transcriptions, tumor profiling, and RNAscope [15-20]. Such molecular analysis of archived specimens will be a significant milestone in research and should be further investigated.

Problems in archiving specimens 

Portions of large specimens are usually left untouched even though adequate sampling was done. A suitable location for archives is underestimated in most institutions. It later leads to inaccessibility, inadequate space, and labor. The lack of a systematic classification of specimens in histopathology, cytopathology, and molecular pathology may add to chaos when in need of specimen retrieval. Adequate qualified personnel such as tissue technologists, administrative staff, and archivists play a coveted role in the pathology setup. Despite regular maintenance, subsequent changes of fixatives are necessary in archives. For specimens requiring particular conditions, such as refrigerated or frozen storage, it is essential to establish suitable measures to guarantee maintenance of the correct storage temperature. This includes implementing emergency protocols in the event of a power supply failure. The temporary storage of "transient" preparations, such as fluorescently labeled cells and tissue sections, as well as other "wet" preparations, should be conducted under appropriate light, temperature, and humidity conditions [9].

At present, there are two ways of specimen storage: in-house archives and outsourced solutions. In-house archives are hospitals or institutions with their archive setup. Outsourced solutions have standard operations procedures with regular documentation, equipment maintenance, and proficiency testing. Financial considerations of maintaining an archive depend on the laboratory size, number, and volume of the specimens to be archived, conservation and functional charges, and the inventories. The associated finance can be divided into direct, indirect, opportunity, and future costs. Ultimately, proper specimen archives must include cost-effective handling and proper storage of specimens, disposal of wastes, infection control measures, and adequate employee training [10,11].

At present, there are no explicit mandates regarding the need for controlled ventilation, lighting, or temperature levels for materials intended for storage under ambient conditions. Guidance in these aspects may develop over time. For instance, in the context of research biobanking involving paraffin wax-embedded tissue blocks (including derivatives such as tissue microarrays), it might be revealed that regulating temperature/humidity becomes crucial for ensuring the long-term preservation of sample integrity [9].

Ethical problems in storing specimens

The ethical, legal, and policy issues affect the researchers’ ability to use the archives besides technical considerations about the quality of a specimen. Using leftover samples demands ethical supervision and patient consent [7]. It again depends on local legislation, as some institutions have a section of the informed consent for treatment that such material is to be used for scientific purposes. Proper regulations must be followed when dealing with human subjects because it may lead to misuse of genetic information [21]. Five major topic areas addressed in the NCI Ethical, Legal, and Policy Best Practices are (a) the principles for responsible custodianship, (b) the informed consent, (c) the privacy protection, (d) access to biospecimens and data, and (e) the intellectual property and resource sharing [22].

Ethical concerns must be addressed by establishing clear guidelines, obtaining informed consent, implementing robust privacy and security measures, and ensuring ongoing communication with patients regarding the use and storage of their biopsy specimens. Ethical supervision by institutional review boards or ethics committees is also crucial in mitigating potential ethical problems in biopsy specimen storage.

Ownership of specimens

Specimens can be obtained from a few sources: (a) tissue collected exclusively for a research project; (b) leftover tissue specimens from samples taken for diagnosis and treatment purposes; (c) tissue specimens after an autopsy; and (d) tissues with reproductive or “human” potential including sperms, eggs, zygotes, fetal tissues, and embryos collected for clinical purposes [23]. When it comes to the leftover tissue specimen, the candidates for ownership could be the patient, researchers, pathologists, institutions, or the laboratory.

The story of Henrietta Lacks and the HeLa cell line started when the leftover tissue samples of the 30-year-old female Lacks, a cervical cancer patient, were passed on to research after diagnosis without her knowledge. The HeLa cell lines are viable even today and gave a great sum of profit to the researchers, but the family received no financial benefits. This was revealed in the book “The Immortal Life of Henrietta Lacks”. The right of investigators and institutions to gather and utilize discarded specimens with related patient data has remained vague and often disputed as laws have grown more stringent and because distinct ownership interests have never been created [3].

Consent

Usually, informed consent does not apply to the research on leftover pathological or diagnostic specimens because the research does not permit or require participant identification. The most agreed opinion is that the consent form describes shortly that the leftover specimen would be stored and could be used for the patient for a second opinion or for a wide range of research applications for which there will be no harm or loss to the patient. Broad consent may not be acceptable for a few individuals who are not willing to use their samples for all types of research. So, those patients who are not willing to use their leftover samples for any future research or any specified future research can always exercise their right not to use their leftover samples for such research [5].

Newly proposed guidelines

**Figure 1:**
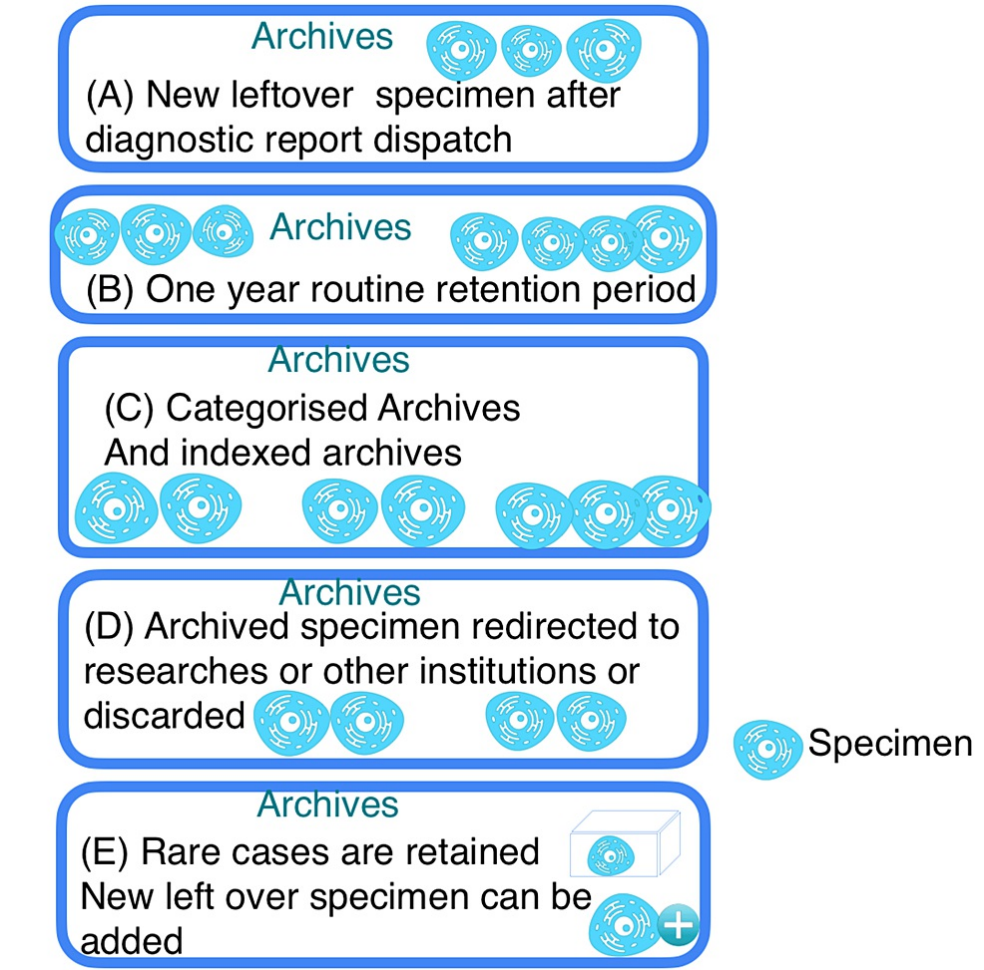
Illustration showing the handling protocol for archive specimens Image credit: N. Fazulunnisa Begum and K. Ramalingam, authors

The protocol can be as follows: (i) Once the diagnostic histopathology report is dispatched, broader consent should be obtained from the patient/attendee stating that the formalin-fixed tissue specimens, blocks, and histopathology slides of the patient would be retained for one year undisturbed (Figure 1A-1B); (ii) After this period, they should be either retained until the stated retention period or used for research purposes. (A retention period of about five years is followed for formalin-fixed tissue specimens, and a retention period of about 10 years is followed for formalin-fixed blocks and histopathology slides.); (iii) The archivists must categorize the specimens, blocks, and slides into histopathology, cytopathology, and molecular pathology, and index them by retention time (Figure 1C); (iv) The archives should be usable by the researcher, with the consent of the pathologist and the institution/laboratory in charge (Figure 1D). Once the retention period ends, the archivists and the pathologists should decide whether to discard the specimens, blocks, and slides to transfer them to other institutions/researchers for teaching purposes or to retain them further, and early disposal and unnecessary longer retention must be avoided; and (v) It is best to retain and archive rare case specimens for longer periods (Figure 1E).

Oral pathology is a specialization in dentistry dealing with diseases in the head and neck region, especially the oral cavity and its associated structures. According to the Dental Council of India (DCI), oral pathology and microbiology are one of the recognized specialties of dentistry [24,25]. According to an international study covering 42 International Association of Outsourcing Professionals (IAOP) member nations, oral pathology is either recognized by a licensing organization or practiced as a specialty. Only 42 of the 195 countries in the globe have oral pathology, while other nations, including Poland and Russia, lack a recognized specialty or licensing organization. Dentists with specialized training in oral pathology diagnose biopsy specimens histopathologically and immunohistochemically, perform fine needle aspiration (FNA) cytology and exfoliative cytology, and analyze biological samples sent for hematology, biochemistry, and microbiology. They treat patients directly or indirectly by coordinating histological characteristics with clinical and radiological findings. Good Clinical Lab Practices (GCLP) encompasses staff training, test procedures, safety, quality control, and data management, fostering excellence in clinical laboratories. GCLP covers every aspect of lab operations, from staff competence to data security, promoting reliable and accurate test results. It did not, however, provide any particular recommendations on oral pathology [26].

Dinesh et al. have reported coverslip removal, destaining, and immunohistochemical assessment of archived slides [24]. Hence, the authors propose these guidelines for the storage and retention of leftover tissue specimens, blocks, and slides. A study by Kokkat et al. showed that there was no significant difference between macromolecules extracted from FFPE blocks stored over 11-12 years, five to seven years, or one to two years compared to the current year blocks [27]. Archival studies have shown immunopositivity for cytoplasmic antigens but poor retrieval of nuclear antigens even after 60 years of storage. Killian and colleagues observed the impressive preservation of high-molecular-weight DNA in archival FNA smears stored for more than 10 years. Despite the lack of uniformity in the collection protocols across the research studies, it appears that they present various alternatives for biobanking FNA specimens. Another suggested method for collecting and preserving FNA specimens for biobanking involves utilizing Whatman FTA (fast technology for analysis (of nucleic acids)) cards (Whatman International Ltd., Maidstone, UK). The FTA card consists of paper infused with reagents capable of lysing cells, releasing DNA that adheres to the card's matrix and remains stable at room temperature. The use of FNA and scrapes has also been reported as appealing choices for biobanking tissue from surgical specimens with small tumors [28].

This study emphasizes the need for defined laws for the management and research of human tissue material by highlighting some of the key features of the subject. It is recommended that institutions adhere to best practices and periodically examine their storage and maintenance policies.

## Conclusions

Specimens are one of the greatest treasures, which are still not utilized to their fullest. They are the tissue reservoirs that can be used for drug development or genetic research using advanced technologies. The usage of these archived specimens can be organized by following standard operating procedures, and this can be initiated by a few guidelines stated in the present study. Further studies and programs should be promulgated for the standard rules for retention and use of leftover specimens: the underestimated treasures. By defining best practices, oral pathologists may be able to collaborate and communicate more with one another, which will raise the standard of care. To the best of our knowledge, there are no systematic reviews or meta-analyses on laboratory administration of oral pathology. There are only a few reported studies on hematology or general pathology. We hope that this review will help future studies on this unexplored area.
